# Give It Up For Baby: outcomes and factors influencing uptake of a pilot smoking cessation incentive scheme for pregnant women

**DOI:** 10.1186/1471-2458-13-343

**Published:** 2013-04-15

**Authors:** Andrew Radley, Paul Ballard, Douglas Eadie, Susan MacAskill, Louise Donnelly, David Tappin

**Affiliations:** 1 Public Health Department, NHS Tayside, Kings Cross Hospital, Clepington Road, Dundee, UK; 2 Institute for Social Marketing, University of Stirling and Open University, Stirling, UK; 3 Health Informatics Centre, University of Dundee, Dundee, UK; 4 Paediatric Epidemiology and Community Health Unit, Child Health, Division of Developmental Medicine, University of Glasgow, Yorkhill, Glasgow, UK

**Keywords:** Smoking, Pregnancy, Smoking cessation, Incentives, Health behaviour, Health promotion

## Abstract

**Background:**

The use of incentives to promote smoking cessation is a promising technique for increasing the effectiveness of interventions. This study evaluated the smoking cessation outcomes and factors associated with success for pregnant smokers who registered with a pilot incentivised smoking cessation scheme in a Scottish health board area (NHS Tayside).

**Methods:**

All pregnant smokers who engaged with the scheme between March 2007 and December 2009 were included in the outcome evaluation which used routinely collected data. Data utilised included: the Scottish National Smoking Cessation Dataset; weekly and periodic carbon monoxide (CO) breath tests; status of smoking cessation quit attempts; and amount of incentive paid. Process evaluation incorporated in-depth interviews with a cross-sectional sample of service users, stratified according to level of engagement.

**Results:**

Quit rates for those registering with Give It Up For Baby were 54% at 4 weeks, 32% at 12 weeks and 17% at 3 months post partum (all data validated by CO breath test). Among the population of women identified as smoking at first booking over a one year period, 20.1% engaged with Give It Up For Baby, with 7.8% of pregnant smokers quit at 4 weeks. Pregnant smokers from more affluent areas were more successful with their quit attempt. The process evaluation indicates financial incentives can encourage attendance at routine advisory sessions where they are seen to form part of a wider reward structure, but work less well with those on lowest incomes who demonstrate high reliance on the financial reward.

**Conclusions:**

Uptake of Give It Up For Baby by the target population was higher than for all other health board areas offering specialist or equivalent cessation services in Scotland. Quit successes also compared favorably with other specialist interventions, adding to evidence of the benefits of incentives in this setting. The process evaluation helped to explain variations in retention and quit rates achieved by the scheme.

This study describes a series of positive outcomes achieved through the use of incentives to promote smoking cessation amongst pregnant smokers.

## Background

Smoking during pregnancy increases the risk of maternal complications, pre-term delivery and low birth weight. Low birth weight is associated with a variety of important adult health problems including coronary heart disease, type II diabetes and obesity [1]. Tobacco smoking during pregnancy is relatively common, especially in low to middle income populations but is strongly associated with poverty, low educational attainment, poor social support and mental health issues [2]. In Scotland, a strong relationship between smoking during pregnancy and deprivation exists, with records at first booking of pregnant women by maternity services showing smoking prevalence rates ranging from 5.8% in the most affluent communities to 29.4% in the poorest, in the year ending March 2009 [3]. In addition, parental smoking in the home has direct, substantial and immediate impacts on children’s health from inhaled environmental tobacco smoke and also potentiates the likelihood of children becoming adult smokers as a consequence of behaviour-modelled parental smoking [4].

There is an emerging body of evidence that suggests financial incentives can be an effective mechanism to encourage smokers to quit [5]. Whilst reward-based programmes have been shown to help initiate and support quitting, it is recognised that to realise their full potential there is a need to develop an understanding of how incentives can be integrated with other forms of structured support in order to sustain the behaviour change [6].

The Scottish Government set a national target to reduce smoking during pregnancy to 20% by 2010 [7]. To help achieve this, women can access smoking treatment services which are free at the point of use in Scotland through the National Health Service. In the intervention health board area (NHS Tayside), uptake of these smoking cessation services by pregnant women was particularly poor [8]. A review of published evidence in the area highlighted that the use of incentives and social support to improve engagement of women who smoked during pregnancy was a promising area to test in practice [9]. Give It Up For Baby (GIUFB) used social marketing techniques to operationalise the evidence available on the role of incentives [10] and was specifically designed to help pregnant women living in deprived communities to stop smoking. The process of social marketing began with an understanding of the requirement to design an intervention that was relevant to the lives of the target audience. This was achieved through initial discussions with community development groups to investigate their ideas and responses to the proposal for an incentive scheme. These groups were used to gauge responses to intervention design and to test publicity materials. In addition, a multidisciplinary stakeholder group was used to ensure engagement of different parties and included representatives from the health service, local authority and community development. The stakeholder group managed the initial implementation process including planning and testing of marketing messages and communications and also gathered feedback from clients and professionals involved in delivering the intervention.

In March 2007, an initial pilot of GIUFB was undertaken in a single urban locality (Area 1) in NHS Tayside Health Board. This area was chosen since a significant proportion of its population experienced substantial levels of social deprivation. The pilot was then rolled out to two neighbouring localities; Area 2 from December 2007 and Area 3 from May 2008, so that the entire health board area was included. These two areas contained a number of rural towns and experienced less social deprivation.

The scheme sought to combine conventional models of behavioural and pharmacological support with a system of financial incentives and social support to encourage women to remain smoke-free [10]. The model was adapted to reflect existing levels of service provision in the three intervention areas; for example Area 2 already incorporated additional cessation support from a local community worker. This paper examines a range of indicators of, and factors associated with, success derived from an evaluation of this service delivery.

## Methods

The methods describe the intervention design and multi-methods evaluation approach which reports findings from two separate data collection exercises; an outcome evaluation based on analysis of monitoring data and a process evaluation with a sub-sample of women who participated in the scheme.

### Intervention design

All women who continued to smoke during their pregnancy in the three intervention areas across NHS Tayside were invited to register with the GIUFB scheme at a local community pharmacy. Women could access information on the intervention from a wide variety of health sources, including through general practices, community pharmacies and ante-natal clinics, as well as through other venues such as local libraries and community centres. In addition local newspapers were frequently invited to publish case examples of women who had successfully engaged with the intervention.

Those women who agreed to take part were asked to set a quit date and received an initial brief intervention and then attended for weekly support from a member of the local pharmacy team for up to 12 weeks. Community pharmacies in NHS Tayside are widely distributed to meet the pharmaceutical care needs of the population and are associated with each community [11]. At the initial meeting, the community pharmacist interviewed the woman and provided free nicotine replacement therapy (NRT) as per assessment. At each subsequent scheduled visit to the pharmacy, the woman was asked to take a carbon monoxide (CO) breath test and received further advice and behavioural support. The CO breath test, as an indirect, cheap and non-invasive measure of carboxyhaemoglobin, was employed in order to give feedback to the women on their progress and to provide a governance trail for managing the intervention process. A CO level of 7 parts per million was used as a cut-off point to validate a non-smoker [12].

The pharmacist communicated the breath test results to the central administrative officer employed by the heath board, who was responsible for managing the collation of weekly results and arranging the payment of the incentive for all negative tests. An incentive payment of £12.50 was chosen arbitrarily for the intervention; little evidence on effective levels of payment was available to inform judgement, although an earlier American intervention used financial incentive vouchers worth $50.00/month [9]. The administrative system enabled participants to be credited with their weekly incentive payment within about 5 working days, which they could then redeem at a local participating supermarket in return for any goods, excluding alcohol and tobacco products. Checkout operators were trained to recognise the scheme’s incentive card and a mystery shopper programme was used to periodically test compliance with the agreed procedures. The scheme was negotiated with the main store in each locality, resulting in partnerships with two major supermarket chains.

Participants who successfully completed the 12 week cessation programme could continue to claim an equivalent monthly incentive up to 12 weeks after the birth of the child by visiting the pharmacy every four weeks and providing a CO breath test. The records of the woman’s attendances at the pharmacy and of the CO breath test provided a primary record of each participant’s quitting behaviour and success.

### Outcome evaluation

Routine monitoring data were used to assess the ability of the GIUFB scheme to support pregnant women to stop smoking. These methods utilised mixed data sources to describe the population of pregnant smokers and to provide an estimate of rates of engagement with GIUFB and quit attempts, including short-term quit rates and longer term smoking cessation outcomes for women giving birth between 2007 and 2010 in the intervention areas.

The denominator of self-reported smoking at first booking with midwifery services is gathered routinely as part of the maternity data collection system which is returned in the Scottish Morbidity Reporting system (SMR02) on an annual basis to the Information Services Division, NHS National Services Scotland [3]. Data on the numbers of live births by locality were obtained from the General Registrars Office for Scotland (GROS) data set for 2009 [13]. Rates of engagement and outcomes for quit attempts were collected through routine data collected by the Public Health Directorate as part of service monitoring. These data were supplemented by data from the National Smoking Cessation Database, which was used to confirm numbers of pregnant women who were recorded as making a smoking cessation attempt within the health board area [14].

The intervention population was defined as all patients who engaged with GIUFB by attending for at least one session at the pharmacy; set a quit date and were entered into the GIUFB dataset held by the health board. As this is an on-going health promotion intervention some patients had not completed the full 12 months at the time of the evaluation. Therefore a cut off date for registration of 31^st^ December 2009 was applied for analysis of outcomes. Results were analysed at 4 weeks, 3 months and for the finishing date of the woman with the scheme, whether that was at 3 months after the birth of the child (completion of the scheme) or if she failed to attend for further tests or provide a positive CO breath test. A woman was assessed as completing the scheme if she visited the pharmacy and provided a CO breath test within 3 weeks of the estimated date of 3 months after delivery of the child.

Data from the health board database were analysed to examine the characteristics of women undertaking the scheme (i.e. women continuing to engage with the scheme and providing negative CO breath tests at four and 12 weeks post quit date and at three months post partum) to assess the effect of the scheme in relation to the number of women from the local population of pregnant smokers who gave up smoking. Descriptive results are presented as N (%) for categorical variables and mean (standard deviation) for continuous variables. Comparisons of patients’ characteristics are by chi-square test for categorical variables and t-test for continuous variables. All p-values are 2-sided. Pairwise comparisons were performed to compare the three intervention areas. In addition, an intention to treat analysis was undertaken for women registering with the scheme in 2009. The analysis was focused on the 2009 period because by this stage, all three areas had established the intervention.

### Process evaluation

The process evaluation was commissioned by the intervention team as a separate service development exercise designed to identify factors that explain differences in client retention with the scheme. For this reason the process evaluation sought the views and experiences of two participant groups with divergent levels of engagement to the service: those who had registered with the scheme but then failed to attend or dropped out typically after one or two consultations; and those who attended on regular basis, often throughout the pregnancy and in some cases beyond.

In this way a cross-sectional sample of 20 service users were identified by local coordinators using client databases to represent the two diverse client groups of interest. Participants were initially approach by a member of the service team to assess initial consent and then followed up by an independent researcher to arrange a time for interview. All data was collected face-to-face in the participant’s home using in-depth interviewing techniques. A cash gift was offered as an incentive and reward for taking part. A small number of interviews were also conducted with local pharmacists (n = 6) responsible for supporting the scheme to provide contextual data on the delivery process. All interviews were conducted in early 2009 with participants drawn from two of the intervention areas who had registered with the scheme in 2008. The number of interviews conducted was restricted by available research resources which were funded from the intervention programme budget.

The interviews were conducted by one researcher using an interview topic guide devised to address a core set of research themes, and revised as the field work proceeded. Minimal cueing was employed to avoid pre-judging or framing interviews to represent a particular position on the scheme. Interviews were digitally recorded with participants’ consent and audio-files fully transcribed and anonymised for thematic analysis.

Analysis was led by the researcher responsible for undertaking the interviews and a core set of themes based on the research questions and topic areas was agreed with another independent researcher. As the analysis progressed, reliability of themes was established via cross-examination and discussion. The transcripts underwent two stages of analysis. Firstly, they were organised using the thematic framework and emerging themes identified through a process of thorough familiarisation with transcript texts. Then the transcripts for all participants were re-read and analysed separately to build a series of individual narratives and case histories. These analyses allowed the investigators to identify patterns across the data as a whole as well as to develop a user typology based on participant profiles and behaviour.

Generalisability of the findings was constrained by two factors: number of interviews and capacity to achieve data saturation limited ability to assess for possible outliers and potential gaps in the typology; and dependence on service engagement as a selection construct limited transferability of findings to other populations of pregnant smokers exposed to different service models. Constraints were also imposed by the multi-methods approach and the extent to which linkages could be established between the various research components. For example, whilst the client monitoring data was used to stratify the sample according to level of engagement with the scheme, there was not scope to integrate the findings of the process evaluation, and in particular the individual user types, with analysis of outcome data. Similarly, governance procedures and the need to maintain participant confidentiality meant the process evaluation was not able to examine interaction between clients and pharmacists at an individual case level.

## Results

### Outcome evaluation

#### Participant characteristics

There were 383 individuals recruited into GIUFB between March 2007 and December 2009. Ten of these women completed the programme twice (subsequent pregnancies), resulting in a total of 393 quit attempts. Table 1 shows the baseline characteristics of the study participants, in total and by area. These data show that the majority of participants lived in areas of high social disadvantage (over two-thirds in Quintiles 1 and 2), reflecting the social demographic structure of smoking in pregnancy and its association with women living in communities of higher social deprivation. Furthermore, 37.9% of mothers were not in paid work. It might be expected that the incentive may have appeared relatively more attractive to some of this group who are less affluent. It is notable that the proportion of women not in paid employment was higher in Area 1 than for Scotland (23% of women in the general population economically inactive in 2010 [15]).

**Table 1:** Baseline GIUFB participant characteristics (registered March 2007-December 2009): total and by area

	**NHS Tayside**	**Area 1 (A1)**	**Area 2 (A2)**	**Area 3 (A3)**	**Pairwise comparisons**^**$**^		
					***A1 vs. A3***	***A1 vs. A2***	***A3 vs. A2***
N	393(100)	160(40.7)	144(36.6)	89(22.7)	0.2041	0.7607	0.3600
Mean age: years (SD)	27.2(6.2)	26.7(6.1)	26.9(6.6)	27.7(5.8)			
*Age groups(years):*							
<20	55(14.0)	26(16.3)	21(14.6)	8(9.0)			
≥20 - <25	110(28.0)	41(25.6)	42(29.2)	27(30.3)			
≥25 - <30	101(25.7)	42(26.3)	39(27.1)	20(22.5)			
≥30 - <40	117(29.8)	49(30.6)	36(25.0)	32(36.0)			
≥40	10 (2.5)	2(1.3)	6(1.5)	2(2.3)	0.4261	0.4321	0.3169
SIMD quintile*:							
1 (most deprived)	142(37.2)	108(67.9)	22(16.1)	12(14.0)			
2	115(30.1)	26(16.4)	57(41.6)	32(37.2)			
3	61(16.0)	7(4.4)	24(17.5)	30(34.9)			
4	41(10.7)	9(5.7)	27(19.7)	5(5.8)			
5 (least deprived)	23(6.0)	9(5.7)	7(5.1)	7(8.1)	**<0.0001**	**<0.0001**	**0.0050**
*Paid employment:*							
Yes	176(44.8)	71(44.4)	67(46.5)	38(42.7)			
No	149(37.9)	59(36.9)	60(41.7)	30(33.7)			
Missing	68(17.3)	30(18.8)	17(11.8)	21(23.6)	0.6528	0.2362	0.0559
*How many cigarettes smoked per day:*							
Less than 10	101(25.7)	2(26.3)	43(29.9)	16(18.0)			
11-20	170(43.3)	65(40.6)	66(45.8)	39(43.8)			
20+	55(14.0)	24(15.0)	18(12.5)	13(14.6)			
Missing	67(17.1)	29(18.1)	17(11.8)	21(23.6)	0.4439	0.3656	**0.0462**
*How long before first cigarette in the morning:*							
Within 5 minutes	126(32.1)	56(35.0)	40(27.8)	30(33.7)			
6 - 30 minutes	96(24.4)	39(24.4)	35(24.3)	22(24.7)			
31-60 minutes	50(12.7)	14(8.8)	28(19.4)	8(9.0)			
After 1 hour	51(13.0)	22(13.8)	23(16.0)	6(6.7)			
Missing	70(17.8)	29(18.1)	18(12.5)	23(25.8)	0.3844	0.0534	**0.0077**
*How many quit attempts in last year:*							
None	141(35.9)	56(35.0)	56(38.9)	29(32.6)			
One	107(27.2)	42(26.3)	43(29.9)	22(24.7)			
2+	71(18.1)	33(20.6)	25(17.4)	13(14.6)			
Missing	74(18.8)	29(18.1)	20(13.9)	25(28.1)	0.2736	0.5867	0.0682

*11 patients with missing data.

Data are N(%) or Mean (SD); Bold values indicate a statistically significant test with P < 0.05; ^$^comparisons by t-test for means and chi-square test for categorical variables.

There were significant differences in social deprivation category across the three areas: approximately 84% of participants in Area 1 were in the two most deprived quintiles (Scottish Index of Multiple Deprivation, SIMD; National Index [16]), compared with 58% in Area 2 and 51% in Area 3. In Area 2, 25% of participants were in the two least deprived quintiles compared with 11% in Area 1 and 14% in Area 3. Pairwise comparison of the three areas also shows that there was a significantly larger proportion of women from SIMD quintile 5 living in Area 1 in contrast to either Area 2 or Area 3.

The average age of women joining the scheme was 27.2 years. Relatively few women from younger age groups engaged, despite high rates of teenage pregnancy, especially in Area 1. The average duration of smoking was found to be 10 years. There were significant differences in the data between the Area 2 and Area 3 participants. Thirty percent of Area 2 participants smoked less than 10 cigarettes a day compared with 18% in Area 3. In addition, 16% of Area 2 participants had their first cigarette more than one hour after waking, a distinct indicator of nicotine dependence [17], compared with 7% in Area 3.

Nearly two thirds of women admitted to smoking more than 10 cigarettes per day. A similar proportion also said that they had not tried to give up previously or had made only one previous attempt in the last year. GIUFB seems therefore to encourage participation, amongst a group who may be otherwise reticent to engage with smoking cessation services.

#### Quit rates

The quit rates for the 393 quit attempts in total and split by region are shown in Table 2. All data were CO validated, reflecting the governance requirement for an acceptable CO test to trigger an incentive payment. Analysis shows rates were 54% at 4 weeks, 32% at 12 weeks and 17%, 3 months after birth. The 4 week quit rate was significantly higher in Area 2 compared with Area 1 (59.7% vs. 47.5%, p = 0.0330) and the 3 month post partum quit rate significantly higher in Area 2 compared with Area 3 (21.5% vs. 10.1%, p = 0.0248).

**Table 2:** Participant quit rates (registered March 2007-December 2009): total and by area

	**NHS Tayside**	**Area 1(A1)**	**Area 2(A2)**	**Area 3(A3)**	**Pairwise comparisons**^**$**^		
					***A1 vs.A3***	***A1 vs.A2***	***A3 vs.A2***
N	393	160	144	89			
4 week pass rate	211(53.7)	76(47.5)	86(59.7)	49(55.1)	0.2531	**0.0330**	0.4833
12 week pass rate	125(31.8)	46(28.8)	52(36.1)	27(30.3)	0.7920	0.1703	0.3657
3 month post partum pass rate	65(16.5)	25(15.6)	31(21.5)	9(10.1)	0.2247	0.1850	**0.0248**

Data are N(%); Bold values indicate a statistically significant test with P < 0.05; ^$^comparisons are by chi-square test.

The 4 week quit rate was compared by deprivation quintile and whether participants were in paid employment or not. The results are presented in Table 3, analysed by locality. Overall, around 47% of those in the most deprived quintile who set a quit date remained quit at 4 weeks. Similar proportions were apparent across all three areas for those in the most deprived quintile, with significantly more participants stopped in the least deprived quintile in the least deprived area (Area 2) compared with the other two areas (Area 1 and Area 3).

**Table 3:** Percentage of participants reaching four week quit mark split by SIMD quintile and paid employment (registered March 2007-December 2009): total and by area

	**NHS Tayside**		**Area 1 (A1)**		**Area 2 (A2)**		**Area 3 (A3)**		**Pairwise comparisons**^**$**^		
	N	N(%) quit at	N	N(%) quit at	N	N(%) quit at	N	N(%) quit at			
		4 weeks		4 weeks		4 weeks		4 weeks			
N	393	211(53.7)	160	76(47.5)	144	86(59.7)	89	49(55.1)	*A1 vs. A2*	*A1 vs. A3*	*A3 vs. A2*
SIMD quintile*:											
1 (most deprived)	142	66(46.5)	108	49(45.4)	22	11(50.0)	12	6(50.0)			
2	115	64(55.7)	26	15(57.7)	57	33(57.9)	32	16(50.0)			
3	61	35(57.4)	7	2(28.6)	24	13(54.2)	30	20(66.7)			
4	41	28(68.3)	9	5(55.6)	27	20(74.1)	5	3(60.0)			
5 (least deprived)	23	12(52.2)	9	4(44.4)	7	5(71.4)	7	3(42.9)	**<0.0001**	**<0.0001**	**0.0074**
*Paid employment:*											
Yes	176	105(59.7)	71	42(59.2)	67	42(62.7)	38	21(55.3)			
No	149	72(48.3)	59	20(33.9)	60	36(60.0)	30	16(53.3)			
Missing	68	34(50.0)	30	14(46.7)	17	8(47.1)	21	12(57.1)	0.0604	0.3960	0.0554

Data are N(%); Bold values indicate a statistically significant test with P < 0.05; *missing for 11 patients; ^$^ comparisons by chi-square test.

A more detailed intention to treat (ITT) analysis of women registering with the scheme in the year 2009 is shown in Table 4. This table demonstrates engagement, retention, and quit rates across the three intervention areas in the context of total pregnant smokers recorded at booking, rather than the denominator of those who engaged with GIUFB. Smoking cessation interventions are associated with significant drop out rates. The ITT approach provides information about the potential effects of the approach on the population of interest, i.e. all pregnant smokers, rather than the effects on the subgroup who engaged more thoroughly in the intervention. The year 2009 was chosen because implementation was fully embedded in all three areas. Table 4 shows that 24.8% of pregnant women overall were recorded as smoking at first booking with midwifery services. This varied from 19.8% in Area 2, the more affluent area, to 28.1% in Area 1, the most deprived area.

**Table 4:** Flow chart for smoking in pregnancy in NHS Tayside: registered January–December 2009

**Parameter**		**Locality**	**Number (% of population)**
1.	Number of Births^a^	Area 1	1754
		Area 2	1361
		Area 3	1168
		NHS Tayside	4283
2.	Number of women smoking at first booking	Area 1	493 (28.1)
	with midwifery service	Area 2	269 (19.8)
	(% of women who gave birth)^b^	Area 3	299 (25.6)
		NHS Tayside	1061 (24.8)
3.	Number of pregnant smokers who make a	Area1	65 (13.2)
	quit attempt with GIUFB^c^	Area 2	74 (27.5)
	(% population who smoke at first booking)	Area 3	71 (23.7)
		CHP^d^ Not Assigned	3
		NHS Tayside	213 (20.1)
4.	Number of pregnant smokers who	Area 1	27 (5.5)
	make a quit attempt and who are	Area 2	33 (12.3)
	successful at 4 weeks	Area 3	20 (6.7)
	(% population who smoke at first booking)	CHP Not Assigned	3
		NHS Tayside	83 (7.8)
5.	Number of pregnant smokers who make a	Area 1	15 (3.0)
	quit attempt and are successful	Area 2	25 (9.3)
	at 12 weeks	Area3	14 (4.7)
	(% population who smoke at first booking)	CHP Not Assigned	1
		NHS Tayside	54 (5.1)
6.	Number of pregnant smokers who make a	Area1	11 (2.2)
	quit attempt and are tobacco-free	Area 2	21 (7.8)
	at delivery	Area 3	10 (3.3)
	(% population who smoke at first booking)	NHS Tayside	42 (4.0)

(^a^ source GROS 2009), (^b^ source ISD 2010), (^c^ Source: Data from Directorate of Public Health NHS Tayside).

Around one fifth of pregnant women identified as smokers engaged with GIUFB, and this varied across localities with 27.5% of the smoking population being recruited in Area 2, compared to 13.2% in Area 1. Overall, 7.8% of pregnant smokers across the three areas had quit at four weeks, with on-going measures showing 5.1% quit at three months and 4% tobacco-free at delivery. Success of women stopping smoking varied across the three localities and reflected the same pattern of engagement. On an ITT basis, approximately twice the proportion of smokers from the population of women admitting to smoking at first booking, were successful at four weeks in Area 2 (12.3%), compared to Area 3 (6.7%). A relatively smaller proportion of women, 5.5%, were successful at 4 weeks in Area 1.

#### Incentive payments

Of the 393 quit attempts 101 (26%) resulted in no payment, primarily because they were smoking after the first week, but also because of participants not continuing with the scheme further than the initial visit. A significantly larger percentage of Area 1 participants received no payment compared to Area 2 (33% vs. 20%, p = 0.0098). The larger number of quit attempts resulting in no payment for Area 1 contributed to the apparent lower mean payment for Quintile 1, since a much larger proportion of Area 1 falls within the SIMD 1 and 2 categories.

### Process evaluation

#### Participant characteristics

Analysis of the participant characteristics indicates average age to be 25.7 years, with the majority living in the most deprived quintile and over half reporting the pregnancy to be a first child. Data on gestational age of the women at engagement was not collected. In accordance with quota selection criteria, equal numbers were recruited from each of the two intervention areas and to represent high and low levels of attendance at a registered pharmacy for advice and CO breath testing (see Table 5).

**Table 5:** Process evaluation participant characteristics

**Area**	**No. of participants**	**Attendance at a registered pharmacy**		**Average age**	**Family status**		**SIMD (V2)**					
							**Quintile**					
							**(Scotland)**					
		Frequent*	Infrequent**		**1**^**st **^**child**	**2 or more**	**Q1**	**Q2**	**Q3**	**Q4**	**Q5**	**DK**
**Area 1**	10	5	5	28.4 years	6	4	8	1	0	0	1	0
**Area 2**	10	6	4	23.0 years	6	4	5	2	1	1	0	1
**All**	20	11	9	25.7 years	12	8	13	3	1	1	1	1

* Quit date set and attended a minimum of 10 consultations with a registered pharmacy at time of interview (average 15.82).

** Quit date set and had either failed to attend or attended a maximum of 5 consultations with a registered pharmacy (average 1.67).

**Table 6:** Client typology type 1 ‘mothers to be’ (n=3)

**Sources**	**Quotes**
C6 (Area 2, Aged 31, 3^rd^ Child, Quintile 1, Frequent attender)	I’d smoked through my previous pregnancies so this time I really, really wanted to give it a bash…
C8 (Area 2, Aged 25, 1^st^ Child, Quintile 2 Frequent attender)	Because I was carrying him, it was for his sake. If I wasn’t pregnant I wouldn’t have stopped, I would have found it really hard.
C8 (Area 2, Aged 25, 1^st^ Child, Quintile 2, Frequent attender)	I was going to do it regardless (of Give It Up For Baby) and it was because you are pregnant you would get free patches… To be honest I only used them (the free patches) for the first two weeks, I knew I didn’t want to harm him (…) But they said ‘well you have to take them, just in case’ – I’ve got boxes and boxes of them at home. I hated the smell of them.
C7 (Area 2, Aged 24, 2^nd^ child, Quintile 1, Frequent attender)	Nobody gets to smoke in here, not anymore. Before I was pregnant and that yeah, people got to smoke in the kitchen, but no, if somebody comes up now they go outside and smoke.
C8 (Area 2, Aged 25, 1^st^ Child, Quintile 2 Frequent attender)	We got loads of nappies, wipes, baby milk, things for him - baby gates and things like that.

**Table 7:** Client typology type 2 ‘novice quitters’ (n=6)

**Sources**	**Quotes**
C20 (Area 1, Aged 22, 1^st^ Child, Quintile 2, Frequent attender)	Everybody was moaning at me so I thought okay, I’ll give it a go, but it didn’t really work. And my mum made a rule that I couldn’t smoke in the house, so I was going outside, which was really hard, but I did it.
C9 (Area 2, Aged 17, 1^st^ Child, Quintile unknown, Frequent attender)	I did like, I tried, but I think I could have tried harder, but like, I think it’s old wife’s tales, all this smoking stuff when you’re pregnant, because she came out at eight (pounds) and there was not one thing wrong with her either, she was a really healthy baby and I think they just over exaggerate, they’re mad on it.
C10 (Area 2, Aged 20, 1^st^ Child, Quintile 3, Infrequent attender)	Interviewer: Did the money figure at all in your thinking?
	Respondent: No, not at all really. I wasn’t bothered. I never got money at the end of it, but I got this voucher through and I was so shocked I even got it cos I thought you had to finish it before you got any money. I didn’t expect to get anything. It wouldn’t have made a big difference cos my mum and dad help me out a lot. They buy nappies every week.
C5 (Area 2, Aged 14, 1^st^ child, Quintile 1, Infrequent attender)	It was my uncle, he was constantly going at me to give up and I just couldn't… I really do want to, but I just, I don't know how to. Anything I try, it doesn't seem to be working.
C5 (Area 2, Aged 14, 1^st^ child, Quintile 1, Infrequent attender)	Interviewer: What about the incentive, does that make any difference?
	Respondent: No, not really. Every week you go, you can change what you're on, you get a week to try it and if you don't like it, you can change it. So, I just did that and I just kept going and changing it and none of them helped me…. I started off with the inhalator, then I went to the patches and then the lozenges and then…
C20 (Area 1, Aged 22, 1^st^ Child, Quintile 2, Frequent attender)	It would have been nice to have someone to speak to because there was nobody else stopping so nobody knew what I was feeling like…. My mum she never smoked in her life, so, she’s like, I can’t believe you’re still smoking, but she has no idea what it’s like to stop (…) If there had been a group of pregnant women and we were all totally in the same boat, that would have been okay.

**Table 8:** Client typology type 3 ‘breadline survivors’ (n=3)

**Sources**	**Quotes**
C16 (Area 1, Aged 19, 1^st^ child, Quintile 1, Infrequent attender)	My sister was pregnant at the same time as me and it was her that told me about it, eh… and said ‘you get money for stopping smoking, like on a card’, and I was like I might give that a go.
C15 (Area 1, Aged 24, 2^nd^ child, Quintile 1, Infrequent attender)	She was okay (the pharmacist), but I think at the same time when I did fall back and I said to her ‘I’ve had one extra than I’m supposed to have today.’ She was kind of like ‘oh no that’s just bad’, she wasn’t sort of sympathetic, if you like. So I just says to her ‘well when you stopped smoking did you not fall back and have one?’ And she said ‘yeah’ and I went ‘well don’t judge me! It was just an accident, a mistake.’
C16 (Area 1, Aged 19, 1^st^ child, Quintile 1, Infrequent attender)	What was important to me was the money really. Cos it could have done so much. With being pregnant and struggling on Job Seekers Allowance with nae top up (…) The money was the thing that actually edged me towards it (the scheme).
C16 (Area 1, Aged 19, 1^st^ child, Quintile 1, Infrequent attender)	The idea sounded really good to me but then when I got the rush to have a fag I just totally forgot all about the money. I really did want to give up but…missing that fag, I think it just wasn’t strong enough. I truly did try and get – I hadn’t like the confidence to do it really. I just don’t think I had what it takes to do it.
C19 (Area 1, Aged 33, 1^st^ child, Quintile 1, Infrequent attender)	I did want to stop smoking and although I wasn’t really a heavy smoker I felt, well I could really do with the £12.50 a week for ASDA tokens that would really be good for me, because I was on benefits… I was on Incapacity Benefit for depression. I’d split up with his father so I was really on my own. I had no support, no money, but it just didn’t really work out all that great.

**Table 9:** Client typology type 4 ‘enthusiastic amateurs’ (n=5)

**Sources**	**Quotes**
C1 (Area 2, Aged 22, 1^st^ child, Quintile 1, Frequent attender)	I know myself I shouldn’t be smoking. If I do slip up and I smoke a fag then I feel so guilty. I’m like, I’m sorry baby and I feel so bad. It’s horrible. It is very difficult…
	It was really easy to cut down when I first started trying to stop. I didn’t find it difficult to cut down. It was going from a couple a day to completely stopping was the hard part. I’d cut down just as I started the scheme I was only on about three or four a day…
C3 (Area 2, Aged 30, 1^st^ child, Quintile 4, Frequent attender)	The only reason I registered was because I knew that I had to stop and by going and registering it would give me an actual date – this is the date and I would say right… Since I’ve been part of the scheme, it has given me that incentive to stop and given me the date, because I was going every week to do the breathalyser thing. (…) I did pretty much do it on my own, but it was worth it to go every week and speaking to the chemist. It gave me that bit of encouragement, ‘Oh you are doing well.’ (…) It was quite good to see the numbers (CO test results) - it was that as well.
C1 (Area 2, Aged 22, 1^st^ child, Quintile 1, Frequent attender)	Interviewer: Has having the money made a difference to you in any way?
	Respondent: It is hard to tell but if the scheme was done without the £12.50 then I probably wouldn’t make as much effort to go to the chemist and speak to Tracy and all this kind of stuff (…) So yeah the £12.50 and being on the scheme does help, it’s been a bit more of a push. It is a bit easier than just trying on your own - It is good to go down and have your test done and be all proud of yourself and say I’m on the GIUFB scheme - But if I wasn’t on the scheme and I wasn’t getting £12.50 I would still be doing it anyway and I’d still be trying my hardest.
C18 (Area 1, Aged 36, 1^st^ child, Quintile 1, Infrequent attender)	For me getting my breath checked – It felt like, if I was to get my carbon monoxide – carbon dioxide measurements – that’s going to give me my incentive – knowing that (I was clear). The money was just a bonus; do you know what I mean?

**Table 10:** Client typology type 5 ‘opportunists’ (n=2)

**Sources**	**Quotes**
C13 (Area 2, Aged 38, 4^th^ child, Quintile 1, Frequent attender)	I’d never thought about giving up smoking. But I mean I didn’t smoke a lot anyway. I wasn’t a heavy smoker. But I just thought with being pregnant I wanted to try and quit it altogether…. It was just a matter of going in, once a week and getting the breath test and seeing how my nicotine level was, and they gave me the patches… But because I wasn’t a heavy smoker, I just kept a hold of the patches and everything; everything just seemed to go, really, really well.
C13 (Area 2, Aged 38, 4^th^ child, Quintile 1, Frequent attender)	Interviewer: What would you have done if the money hadn’t been available?
	Respondent: I would have gave it up for her. I just think because the money was there it was – it was a way of making me stronger…
	Interviewer: You’d not even had a wee lapse?
	Respondent: Nah! Nothing! Nothing! No once I’d given up, I’d given up and that was it. I only needed the patches for quite a short period of time… There was sometimes when I forgot to put it on, you know? And I didn’t even notice. The money wasn’t like a giant (incentive)…but that was why we took it, we took it because, yeah it’ll help get everything for her, that was the reason we took the incentive because of the money for her.
C17 (Area 1, Aged 22, 1^st^ child, Quintile 1, Frequent attender)	I stopped, because I knew I was pregnant, I was wanting to do everything sort of right. And then I heard about this and that made us more determined as well like. (…) I was determined to stop smoking at the time and that’s what it was, just one day I just said right we ain’t smoking, and we never smoked.

**Table 11:** Client typology type 6 ‘impulse shoppers’ (n=1)

**Sources**	**Quotes**
C2 (Area 2, Aged 26, 5^th^ child, Quintile 1, Infrequent attender)	There has been a few attempts, a lot of attempts and the first few times it was only like a week or so I managed… but I did want to give up, I wanted to give up when I was pregnant with her but I just, I don’t know, I just couldn’t (…) Sometimes the doctors kind of, they kind of look down their nose at you and it’s like “Oh you’ve been here before, you never managed you know, the past three, four times, what makes you so sure you’re going to manage now?”, and they don’t have much compassion I suppose the word is.
C2 (Area 2, Aged 26, 5^th^ child, Quintile 1, Infrequent attender)	I was at the chemist seeing if I could get patches without having to go to the doctor and she took me in to this little room and gave me loads of leaflets and one of them was about this baby thing, I wasn’t really too bothered about it but she put my name down and everything anyway because I wasn’t sure at the time whether I’d be able to stick to it or not so, but that’s how I heard about it. (…) She asked me if I wanted to go for it or not and I kind of felt obliged to because I was just in this wee room and there wasn’t much going on, there was all these leaflets in front of me and I just kind of, I mean I only went in there to get patches and I was getting hit with all this stuff and I was like ‘Just put my name down, whatever’.

#### Client typology

Participant data and case histories were used to develop a client typology to help explain variations in client retention and how the scheme benefited particular user groups. A synopsis with supporting quotes is provided for the six user types to emerge from the case analysis. These illuminate defining characteristics for each user group such as: age, social circumstances and experience of parenthood; attitudes to smoking during pregnancy and motivation to quit; circumstances surrounding the decision to sign up to the scheme; values attached to different aspects of support including the role played by the incentive; and benefits derived from the scheme and implications for the decision to continue to attend for support.

**Type 1: Mothers to be (see Table **6**for illustrative quotes)** For this group the child and the child’s health were at the forefront of their decision-making. These were often first time mothers who had always assumed that pregnancy would be the big test; a time that they would make a serious attempt at stopping. The group also included some second time mothers who expressed feelings of guilt and wanted to “do it right” this time round. Overall motivation to quit, and in some cases, confidence, were high and quitting aids tended not to be particularly important. Some used NRT, while others expressed concerns about damage to the child. Some also introduced strict smoke free restrictions in the home and abstained from drinking as well. Most in this group were frequent attenders.

**Type 2: Novice quitters (see Table **7**for illustrative quotes)** These were typically younger, dependent first time mothers still living within the family home. They had limited motivation and were looking for a simple solution. Consequently, NRT aids were often their primary interest and incentives were of limited value because they were still dependent on parents for financial support and maternal instincts could be limited due to lack of maturity. This emerged as the group least likely to question the health risks of smoking to the unborn child and was the group that expressed least support for the scheme, with many coerced to register by close family and health workers. They appeared to require more intensive, tailored social support and were open to group therapies and meeting with other mums in a similar position.

**Type 3: Breadline survivors (see Table **8**for illustrative quotes)** These were mothers who appeared to be the most socially and financially disadvantaged, typically single mothers and/or with unsupportive partners living in impoverished circumstances. They tended to place a particularly high value on the financial rewards offered by the scheme which were more likely to be used for buying staples such as groceries rather than baby products or treats. Mothers in this group tended to have relatively low self-esteem and confidence in their ability to stop and were also the group whose relationships with the pharmacists were more likely to be characterised by conflict, with CO test results sometimes contested. Many described relapsing on a number of occasions and most were infrequent attenders at their registered pharmacist.

**Type 4: Enthusiastic amateurs (see Table **9**for illustrative quotes)** Most in this group had a history of quit attempts which were often unplanned, and poorly organised. They required (and in some instances looked for) structure to provide the necessary discipline and focus for initiating a successful quit attempt. For many in this group the CO test result was often, as important, or more important than the financial incentive to maintaining the quit attempt. Many had cut down their smoking before coming to the scheme but struggled to get to zero. Women in this group ranged from young first-time mothers to older mothers with existing children who had tried but failed in the past. This was the group that appeared to benefit most from the scheme, often describing how the financial component and the positive reward of passing the CO breath test combined to provide the incentive to routinely attend. NRT and informal social support from pharmacist were also important to this group.

**Type 5: Opportunists (see Table **10**for illustrative quotes)** These were light smokers, relatively confident of their ability to give up unaided. In view of this they saw the scheme as a financial opportunity and often conceptualised it in material terms as a benefit or means of supplementing the amount of money they had for buying baby products. Given the relative ease with which they stopped smoking, a defining characteristic of this group was their relationship with the scheme, which was largely perfunctory, with little value being attached to the pharmacist or what s/he had to offer in terms of emotional and physical support.

**Type 6: Impulse shoppers (see Table **11**for illustrative quotes)** This type included people with limited commitment, drawn into the scheme when making speculative enquiries for quit advice. They appear to be mothers who had tried to quit numerous times in the past but failed. They also tended to have low self-esteem and did not expect to succeed, but believed they should try; ‘pregnant again’.

## Discussion

Engagement of women who continue to smoke during their pregnancy is challenging, especially in deprived areas, with 30% of pregnant women smoking at booking in most deprived areas in Scotland compared to 6.7% in least deprived [7]. Give It Up For Baby aimed to encourage engagement and cessation through the offer of financial incentives in NHS Tayside health board area. Quantitative data for this evaluation was derived from routinely collected data used in the administration of the scheme. A formal control area was not established as GIUFB resulted from service development rather than a research programme. However, work to review the effectiveness of existing smoking cessation services for pregnant women across Scotland provides useful comparisons with the results of this study [18]. Tappin et al. analysed data and reported uptake and successes in 2006 based on all pregnant smokers across Scotland living in areas with recognised specialist or good generic services (rather than rates for those already engaged with services). These data covered all existing services for pregnant women. Whilst these services had evolved with differing configurations, ranging from opt-in intensive home based support to opt-out clinic based services; none incorporated financial incentives or required weekly attendance at pharmacies for CO readings. These can be compared with the GIUFB data similarly based on all pregnant smokers reported at the booking visit for 2009 (Table 4). Tappin et al. found that only a small proportion of pregnant smokers (as reported at the booking visit) were supported to stop across Scotland, with only 13% of pregnant smokers having engaged and set a quit date in 2006, compared with 20.1% engaging and setting a quit date with GIUFB in 2009. Furthermore, only 3.9% quit smoking during pregnancy at 4 weeks in 2006 across Scotland, from an intention to treat viewpoint. In contrast, the GIUFB intervention supported 7.8% of pregnant smokers to quit at 4 weeks across the three intervention areas (range 5.5%, Area 1 to 12.3%, Area 2). The nearest comparative performances in Scotland were 5.0% for an opt-out clinic based service and for an opt-in home-based service [18]. The GIUFB outcomes therefore indicate a substantial improvement on national figures in relation to engagement and cessation. Of note, however, are the differences between rates of engagement and quit success within the three intervention areas, with a higher proportion of pregnant smokers from the more affluent area (Area 2) engaging and quitting at 4 and 12 weeks than women from the substantially more disadvantaged locality (Area 1).

It is notable that results reported for GIUFB by community pharmacies were based on weekly CO breath testing. The results from GIUFB are therefore likely to be more reliable than self-reports of tobacco abstinence in other datasets. For example, in the review of services in Scotland, only half of quits were CO breath tested [18]. In addition, this aspect of the scheme was valued by participants, as it gave immediate feedback and provided a focus for a behavioural support discussion. Although CO breath-testing has limitations as a tool for validation of smoking status, especially in terms of the persistence of CO on the breath, the method was simple to administer and had face validity for participants. Use of other methods such as salivary or blood testing of cotinine were felt to have significant drawbacks because of their invasive nature [19].

Focussing on GIUFB participants 2007–2009, rather than all pregnant smokers in Tayside, the GIUFB intervention was also able to demonstrate that 53.7% of women managed to continue their quit attempt for 4 weeks. This is higher than in the earlier review of Scottish services [18] which found that 29%-35% were successful at 4 weeks post-quit date, depending on service type. The 4 week success rate for GIUFB was highest in the more affluent intervention area at 59.7%, a substantial increase on the standard Scottish performance for four week quits (38%). This pattern is also repeated for 12 week quits where GIUFB achieved a 31.8% quit rate compared to a 15% 12 week quit rate reported by the NHS Smoking Cessation Service statistics [14]. Smokers are 1.5 times as likely to set a quit date (relative risk (95% CI) of 1.58 (1.38-1.81)) and twice as likely to be quit 4 weeks later with GIUFB compared with other specialist smoking cessation services in Scotland where incentives are not part of the intervention strategy (relative risk (95% CI) of 2.03 (1.60-2.59)) [18].

Interestingly, while GIUFB utilised community pharmacies as the service delivery route, in general, community pharmacies across Scotland deliver a lower 4 week quit rate than other smoking cessation service settings [14]. The evaluation of a similar smoking cessation incentive scheme (Quit4U) has suggested that the weekly CO breath test provided a place from which to engage the participant in a supportive discussion [20]. A further paper evaluating this incentive scheme proposed that the smoking cessation incentive scheme might reverse the felt contractual relationship between service-provider and client with the client now the provider who is paid to quit [21].

Although the literature suggests it is likely that incentive schemes may encourage engagement, the effectiveness of incentive schemes in promoting compliance in complex behaviour change is still challenged [6]. This paper presents some further evidence that using incentive schemes to promote smoking cessation in pregnant women through NHS service provision can also improve and maintain the patient outcomes that are delivered. For example, across the three years of implementation of GIUFB, 16.5% of women engaged with GIUFB have been found to be abstinent at three months post partum. Further challenges to the use of public money for incentive schemes have been made and little evidence exists to gauge the correct level of incentive to produce a worthwhile effect, although NICE have recommended small cash amounts for contingency management in substance misusers [22]. Some emerging evidence suggests that the beneficial effect of the incentive may be sustained beyond the period of payment: the Quit4U intervention mentioned above provided incentives for 12 weeks and the evaluation showed 46% increase in cessation at 12 months compared to the Scottish benchmark [20].

A critical issue to emerge from the outcome evaluation was differences in the level of engagement and quit success achieved in the different intervention areas, with the service performing better in the more affluent area. This is particularly pertinent given similar patterns were also found by the process evaluation and the difficulties experienced in tackling health inequalities by targeting disadvantaged communities [23,24]. Data from this study appear to suggest that women from more affluent communities derive greater benefit from the incentive scheme than those from more deprived communities. In addition, older women and women undertaking a second pregnancy were more likely to engage, with young smokers less likely to engage. The reason for this may be partially explained by service-level factors with, for example, more effective support in the more affluent area being provided by a peer support worker and midwives in the same area being better positioned to forge closer relationships with their clients due to fewer competing priorities and pressures on time. Nevertheless, 4 week quit rates of over 45% in the most deprived quintile represents a substantial achievement, as does engagement by 20.1% of the women still smoking at their booking visit.

The process evaluation conducted with a small sample of participants was used to develop a service user typology which provides additional insight into why some women benefited more from the scheme than others. This is the first time such a typology has been developed and whilst the sample size places constraints on the generalisabilty, these findings were particularly helpful in shedding light on why socio-economic factors appears to predict level of engagement with the scheme and differences in quit rate. Self-reported smoking behaviour from the process evaluation indicated that those least successful at quitting tended to be ‘breadline survivors’, often single mothers who as a consequence of their deprived circumstances and competing lifestyle pressures had less commitment to stopping and who attached a greater value to the material incentive than to the cessation support on offer. In contrast those who were more successful tended to conceptualise the incentive as part of a wider reward structure (e.g. ‘mothers to be’ and ‘enthusiastic amateurs’). In these groups the financial reward appeared to help to incentivise continuing, routine participation and engagement with the support on offer, rather than quitting per se. For these participants other aspects of the scheme were equally as important, for example, confirmation of their non-smoking status delivered by regular CO breath tests, and the supportive relationships established with their local community pharmacy. In addition, the process evaluation also found that younger smokers or ‘novice quitters’ who were still living in the family home and dependent on parents for support, attached a low value to the material incentives and were relatively unsuccessful at quitting. Findings suggest that these smokers express feelings of isolation and report being open to the idea of meeting with other pregnant mothers in a similar position as themselves. There was also evidence that for some successful quitters the material incentive had limited effect, especially for light smokers, or ‘opportunists’ who reported being able to give up without support and would have done so irrespective of participation in the scheme.

Variations in self-reported quit rates to emerge from the process evaluation interviews were also supported by attendance levels at registered pharmacies and CO breath test data. Taken together these findings provide some valuable insight into how incentives work with different sub-groups and suggest that there may be scope to develop these typologies to help identify and target those user groups more likely to benefit from approaches incorporating a financial incentive. Difficulties in linking the process and outcome data also highlights the importance of a mixed methods approach to data collection incorporating an integrated research design. Such an approach does not typically fall within the scope of service development programmes and requires additional resources and planning.

## Conclusions

This study has shown that GIUFB, which provided financial incentives via community pharmacy support and CO monitoring for pregnant smokers, achieved higher engagement rates and higher quit rates than other non-incentive based pregnancy interventions in Scotland. Whilst pregnant mothers from less disadvantaged areas derived most benefit, the scheme enabled substantial reach to those in more disadvantaged circumstances and made a modest contribution to reducing health inequalities, since although lower quit rates were achieved by smokers from areas of higher deprivation, the proportion of smokers attempting to quit was higher. Variations in successes and the factors associated with these outcomes act as useful drivers for guiding programme development. This study demonstrates the practical application of incentives within a heterogeneous population and delineates some of the variation in participant response. Some insights into the variability in the client group and the differential benefit derived by populations living within disadvantaged communities is also shown.

## Competing interests

AR and PB are employed by NHS Tayside and have a coordinating role with regard to the scheme. SM, DE, LD and DT have no interests to declare.

## Authors’ contributions

AR, DE and SM contributed to the design of the evaluation. LD and AR analysed the routinely collected data. DE analysed the qualitative interview data. All authors contributed to the interpretation and writing of the paper and have read and approved the final manuscript.

## Pre-publication history

The pre-publication history for this paper can be accessed here:

http://www.biomedcentral.com/1471-2458/13/343/prepub
